# Fertility and contraception among women of reproductive age following a disaster: a scoping review

**DOI:** 10.1186/s12978-022-01436-4

**Published:** 2022-06-23

**Authors:** Penelope Strid, Margaret Christine Snead, Romeo R. Galang, Connie L. Bish, Sascha R. Ellington

**Affiliations:** Division of Reproductive Health, Centers for Disease Control and Prevention, 4770 Buford Highway NE, Mailstop S 107-2, GA 30341 Atlanta, USA

**Keywords:** Fertility, Contraception, Disaster, Review

## Abstract

**Background:**

The prevalence and severity of disasters triggered by natural hazards has increased over the last 20 years. Women of reproductive age may encounter unique reproductive health challenges following a disaster. In this scoping review we identify gaps in literature to inform future research and search for potential associations between disasters by natural hazards and post-disaster fertility and contraception among women of reproductive age.

**Methods:**

Medline (OVID), Embase (OVID), PsycInfo (OVID), CINAHL (Ebsco), Scopus, Environmental Science Collection (ProQuest Central), and Sociological Abstracts (ProQuest Central) were searched for articles published from 1980 through March 3, 2022 in English or Spanish language. Search terms were related to fertility, contraception, and disasters. We included original research that described a discrete natural hazard exposure, a population of women of reproductive age (15–49 years), and outcomes of fertility or contraception use or access, with pre- and post-disaster measures.

**Results:**

Among 9788 citations, after initial exclusion 5121 remained for title and abstract review. One hundred and eighteen citations underwent full-text review and 26 articles met the inclusion criteria. Following critical appraisal, 20 articles were included in this review. Eighteen articles described outcomes related to fertility, five articles described contraception access, and three articles described contraception use.

**Conclusions:**

Clearly defined exposure measures, robust analyses, and methodical post-disaster assessment periods, may address the current gaps within disaster research on fertility and contraception among women of reproductive age. Consistent patterns in fertility following a disaster triggered by natural hazards were not identified between or within disaster types. Studies that assessed contraception found no change in use, while some studies found a decrease in contraceptive access overall.

**Supplementary information:**

The online version contains supplementary material available at 10.1186/s12978-022-01436-4.

## Plain English Summary

Natural disasters are becoming more frequent and severe. In this scoping review, we explore published literature from 1980 to March 3, 2022 on the impacts of natural disasters for women of reproductive age, 15–49 years. We assess gaps in the literature and search for possible trends in fertility and contraception use and access after a disaster. A targeted literature search in multiple databases resulted in 9,788 citations. Systematic methods were used to identify relevant articles for this scoping review. Of the 20 articles included, we identify several gaps. Future research may benefit from improved disaster exposure measurements, comparing exposed samples to a similar unexposed sample, and measuring outcomes at purposeful post-disaster time points. No consistent patterns were identified among studies assessing post-disaster fertility. Contraception use did not appear to change following disasters, while contraception access generally decreased.

**Supplementary information:**

The online version contains supplementary material available at 10.1186/s12978-022-01436-4.

## Introduction

Disasters can be triggered by natural hazards such as earthquakes, hurricanes, floods, tsunamis, and wildfires threatening substantial damage to property and human health. The frequency and severity of these types of disasters have increased over the last 20 years, affecting more than three billion people worldwide [1]. While challenges for whole communities may vary by disaster hazard type and severity, women of reproductive age (WRA), 15–49 years, are at unique risk for negative impacts to their reproductive health following a disaster [2].

A 2012 systematic literature review [2] examined reproductive health outcomes among WRA following disasters in the United States and identified three studies describing fertility after a natural hazard disaster. Results were mixed; disaster exposure was associated with increased fertility in one study [3] and decreased fertility in two studies [4, 5]. Additional studies have since been published using various data sources and report changes in fertility associated with disasters [6–8]. Factors affecting fertility after a disaster are unclear, but may include increased interpersonal conflict, uncertain economic conditions, changes in pregnancy desires and plans, as well as changes in access to and use of contraception [3, 6, 8, 9]. After a disaster, changes in contraception use may vary based on accessibility, supply, and demand [7, 10]. For example, changes to contraception access may result in couples changing to a less effective method and lead to unintended pregnancies [11]. Contraception use may be altered if fiscal and economic resources are impacted following the disaster, and post-disaster stress may alter contraceptive use adherence, decreasing efficacy [3, 12]. During emergency relief in the post-disaster period, the prioritization of contraceptives may be lacking [12, 13]. Understanding fertility and contraception use and access in the post-disaster setting can inform emergency preparedness and response planning and better support people in their reproductive life plans following a disaster.

Our scoping review updates and expands upon the search criteria used by Zotti et al. [2] in their 2012 review. We summarize available literature regarding the impacts of disaster caused by a natural hazard for WRA on fertility and contraception use and access. We identified gaps in the literature to inform future research and searched for potential associations between exposure to disasters and the outcomes of fertility and contraception use and access.

## Methods

### Search strategy

This review was developed according to the Preferred Reporting Items for Systematic Reviews and Meta Analyses Scoping Review extension checklist [14]. Preliminary searches showed no evidence of literature available on these topics in the context of natural hazard disasters prior to 1980. Medline (OVID), Embase (OVID), PsycInfo (OVID), CINAHL (Ebsco), Scopus, Environmental Science Collection (ProQuest Central), and Sociological Abstracts (ProQuest Central) were systematically searched for articles published from 1980 through March 3, 2022 in English or Spanish. Search terms were related to fertility, contraception, and disasters (Table 1). Citations of all articles selected for study inclusion were reviewed for additional relevant articles.

**Table 1:** Medline (OVID) search strategy

disasters/ OR disaster planning/ OR strategic stockpile/ OR mass casualty incidents/ OR medical countermeasures/ OR exp Natural Disasters/ OR exp Climate Change/ OR (natural disaster* OR public health emergenc* OR climate change OR global warming OR (extreme ADJ2 weather) OR (extreme ADJ2 temperature*) OR (extreme ADJ2 heat) OR earthquake* OR drought* OR flood* OR hurricane* OR storm OR storms OR tornado* OR (volcan* ADJ2 erupt*) OR wildfire* OR wild fire* OR terrorist* OR bioterror*).ti,ab.
AND
Pregnant Women/ OR Pregnancy/ OR pregnancy, unplanned/ OR exp contraception/ OR exp pregnancy complications/ OR Abortion, Spontaneous/ OR (pregnant OR pregnanc* OR contraception OR contraceptive* OR Plan B OR IUD* OR condom* OR LARC OR birth control OR family planning OR abortion* OR reproductive health OR reproductive age OR fertility OR birth rate* OR births).ti,ab.
Limit to English and Spanish; 1980 -; Abstract available

### Study selection

An initial review removed duplicate citations and citations with a non-human population, an infectious disease outbreak, or an exposure of humanitarian crisis related to conflict. Two blinded reviewers screened the title and abstract of remaining citations using RAYYAN software (Qatar Computing Research Institute) [15]. Discordant review determinations were reconciled by a third reviewer. Citations meeting the following inclusion criteria were included for full-text review: non-review article and had an exposure of a disaster or extreme weather event, a population of WRA, and outcomes related to pregnancy or contraception.

During full-text review, articles were assessed for: an exposure limited to disasters describing a discrete event, excluding periods of extreme weather (e.g., drought); a population of WRA; and outcomes related to fertility and contraception use or access. Articles published in journals as original research were included while other publication types including abstracts, commentaries, conference proceedings, dissertations, opinion pieces, and reviews were excluded. Studies without pre- and post-disaster measurements were excluded, as this review aimed to describe patterns of association between the disaster and outcomes.

### Data abstraction

Data were abstracted using a Microsoft Access 2016 form created for this scoping review (Additional file 1). Full-text review and data abstraction methods were standardized across reviewers using a 10% sample of randomly selected citations, which underwent full-text review and group discussion by the entire author group. Full-text review and data abstraction were performed in duplicate. Discrepancies between the two full-text reviewers were resolved by the entire author group. The study design for all citations undergoing full-text review was recorded, along with a decision to include or exclude. Exclusion reason was assigned using the following hierarchy: wrong exposure, wrong population, wrong publication type, wrong outcome, or wrong study design. The following information was abstracted from included articles: location of disaster, study population, sample size, length of follow-up, type of disaster (e.g., earthquake, hurricane, flooding, tsunami), fertility outcomes (e.g., birth rate, total fertility rate, monthly hospital births), and contraception outcomes (i.e., access and use). When birth and population counts were available, birth rates per 1000 population per year were calculated.

### Critical appraisal

All included articles underwent a critical appraisal by two reviewers using the National Heart, Lung, and Blood Institute quality assessment tool for observational cohort and cross-sectional studies [16]. Definitions for quality ratings of good, fair, or poor were agreed upon by all authors prior to conducting critical appraisal. Articles deemed poor quality were excluded from further analysis (Additional file 2).

## Results

### Search results

**Fig. 1:**
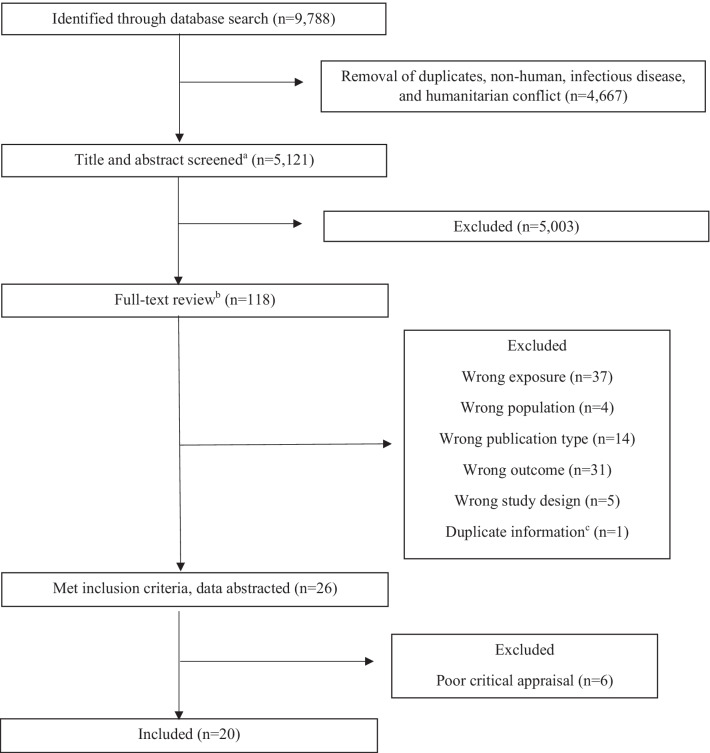
Flowchart summarizing literature search and selection process for scoping review. ^a^Titles and abstracts were screened for non-review articles with a disaster or extreme weather event expsoure, a population of women of reproductive age (15–49 years), and outcomes related to pregnancy or contraception. ^b^Full-texts were reviewed for original research describing a discrete natural hazard disaster exposure, a population of women of reproductive age, and an outcome of fertility or contraception use or access, with pre- and post-disaster measures. ^c^Excluded Harville [17] for duplicate reporting without additional information compared to Hamilton [5]

Database searches yielded 9788 citations (Fig. 1). After an initial exclusion, 5121 citations remained for title and abstract review. We completed full-text review on 118 citations. Ninety-two citations were further excluded. Thirty-seven citations were excluded due to wrong exposure (e.g., the study exposure was not a discrete disaster of natural hazard). Four citations were excluded due to wrong population (e.g., the study population was not WRA). Fourteen citations were excluded due to wrong publication type, and 31 citations were excluded due to wrong outcome (e.g., the studies did not assess fertility or contraception). Five citations did not describe pre- and post-disaster measurements and were therefore excluded. One citation was excluded for duplicate information as it described a sub-set of data included in another report [17]. Twenty-six articles remained for critical appraisal. Six articles received a quality rating of poor, leaving 20 articles for inclusion in this scoping review.

### Study characteristics

Among the 20 articles included in this scoping review, the studies included exposure to disasters (earthquake, n = 10; hurricanes, n = 7; tsunami, n = 2; and flood, n = 1) occurring between 1989 and 2012. The number of years from disaster event occurrence to study publication varied from one to 19 years. Multiple disasters were described by two articles; the 2004 Indian Ocean Tsunami [8, 18], hurricanes occurring in Florida in 2004 [19, 20], Hurricane Katrina in 2005 [5, 6], the 2010 Chile Earthquake [21, 22], and the 2011 Great East Japan Earthquake [23, 24]. Ten studies used a cohort study design, eight studies performed an analysis of longitudinal administrative data, and two used mixed methods including interview. Thirteen articles described a disaster occurring outside of the United States (i.e., Chile, China, Haiti, India, Indonesia, Iran, Japan, Nicaragua) and seven described exposure to a disaster occurring within the United States (i.e., Alabama, Florida, Louisiana, Mississippi, North Dakota, South Carolina). Exposure to disaster events were generally defined by the affected geographical area, and in some cases measured by rainfall, wind speed, storm advisories, and federal disaster declarations. Hurricane exposure was categorized by wind speed, distance from storm path, storm advisories and warnings, and Federal Emergency Management Agency disaster declarations. Grabich et al. [19] compared results using two exposure measures, wind speed and storm path, and came to similar conclusions. Evans et al. [25] used storm advisories and warnings, suggesting behaviors change when storm projections are released, regardless of the storm’s actual path. Eighteen of the included articles described outcomes related to fertility (e.g., birth count, birth rate, fertility rate), five described outcomes related to contraception use or access (e.g., report of contraception use, report of unmet need for contraception, access to condoms, and change in contraceptive method), and three described outcomes related to both fertility and contraception use or access (Table 2).

**Table 2:** Summary and characteristics of 20 articles assessing fertility or contraception following natural hazard disasters 1989–2012

First author (year)	Study design and time period assessed	Disaster location, type and date	Sample	Exposure measure(s)	Outcome(s) assessed	Key findings	
Bahmanjanbeh (2016)	Cohort Pre-disaster: 1 year before, 2011 Post-disaster: 1 year after, 2013	Earthquakes August 12, 2012 East Azarbaijan, Iran	All married women 15-49-years-old living in earthquake affected area n = 44,265	6.3 and 6.4-magnitude, Richter scale—All births after earthquake were considered exposed	Fertility Contraception access	Birth Rate (per 1000 population/ year) 1 year before: 18.5 Year of disaster: 18.3 1 year after: 17.8 Marriage Fertility Rate 1 year before: 111.7 Year of disaster: 109.1 1 year after: 103.2 Contraceptive Coverage (%) 1 year before: 66.9 Year of disaster: 66.8 1 year after: 64.9	
Behrman (2016)	Cohort Pre-disaster: 5 years before, 2005 Post-disaster: 2 years after, 2012	Earthquake January 12, 2010 Haiti	Population survey of women 15–49-years-old Pre-disaster: n = 10,757 Post-disaster: n = 14,287	4.61–7.65, Mercalli score—Period after earthquake considered exposed Compared department-level destruction by Mercalli score	Contraception use and access	Contraception Use Difference-in-Differences (DID) suggests there is no significant effect on the probability of using a modern contraception method Contraception Access Significant** increase in an unmet need for contraception.	
Cohan (2002)	Longitudinal administrative 1975–1997	Hurricane September 22, 1989 South Carolina, US	Population vital statistics for state and counties	Category 4 Hurricane—Severity determined by federal disaster declaration, and seven most severely affected counties were first reported disaster declarations. Compared to 22 counties in South Carolina without federal disaster declaration	Fertility	Birth Rate (per 100,000 population/year) 1 year after: Net increase of 41 In the year following the hurricane, counties with a federal disaster declaration had a significant* increase in birth rate compared to counties in the state that were not declared disaster areas.	
Davis (2017)	Cohort Pre-disaster: April–September 1998 Post-disaster: 2 years after, August 1999–July 2001, and 5 to 7 years after, November 2003–October 2005	Hurricane October 28, 1998 Nicaragua	Women 15–49-years-old residing in zones where precipitation occurred from hurricane Pre-disaster: n = 5424 Post-disaster: August 1999–July 2001 n = 5353 November 2003–October 2005 n = 8734	Category 5 Hurricane—Compared mean rainfall level per municipality during the 10-day storm period of the hurricane	Fertility	Total Fertility Rate All women 1998: 3.01 2001: 2.81 2005: 2.75 Women in zones with below median precipitation 1998: 3.41 2001: 3.27 2005: 3.02 Women in zones with above median precipitation 1998: 2.62 2001: 2.36 2005: 2.36	
Djafri (2015)	Mixed methods Population statistics: 2007–2011 Health facility-based review: conducted November 2010–May 2011	Earthquake September 20, 2009 Padang, Indonesia	Population statistics of Padang City Women 15–49-years-old receiving service at local health center at least twice before earthquake	7.6-magnitude, Richter scale—Period after earthquake considered exposed	Fertility Contraception use and access	Birth Rate (per 1000 population/ year) 2007: 17.0 2008: 18.3 2009: 18.8 2010: 19.8 2011: 19.6 Contraceptive Use—No change Contraceptive Access—Perceived ability to access contraception declined by 20% for 1–3 months after	
Evans (2009)	Longitudinal administrative 1996–2002	Hurricanes Gulf Coast Region, US	Population vital statistics for states and counties	Storm advisories	Fertility	Number of births—Change in monthly county births compared to prior year, same month Tropical storm watch: 3.2% decrease 10 months after**, 2.6% increase 11 months after* Tropical storm warning: Constant Hurricane watch: 2.6% increase 10 months after**, 3.7% increase 11 months after**, 0.9% increase 3 years after* Hurricane warning: 2.2% decrease 9 months after**, 2.6% decrease 10 months after**, 0.7% decrease 3 years after*	
Grabich (2015)	Cohort Pre-disaster: August 14, 2003–October 31, 2003 Post-disaster: 2004	Hurricanes August 13, 2004 and September 21, 2004 Florida, US	Conceptions resulting in live birth among Florida female residents 15–45-years-old n = 92,398	Wind severity in county (≥ 74 mph), and county distance from storm path (< 60 km)	Fertility	Birth Rate (per 1000 population) DID—No association observed between hurricane exposure and birth rate GLM—Risk difference of 2.2 births per 1000 population (95% CI: 1.5, 3.0) when wind speeds are ≥ 74 mph compared to < 74 mph. Risk difference of 2.8 births per 1000 population (95% CI: 1.9, 3.7) in storm path compared to those outside 60 km buffer of storm path	
Grabich (2017)	Cohort January 2003–October 2004	Hurricanes August 13, 2004; September 5, 21, and 25, 2004 Florida, US	Conceptions resulting in live birth among Florida female residents 15-45-years-old n = 138,005	County exposure to hurricane weather conditions, and by wind strength (≥ 39 mph, ≥ 79 mph)	Fertility	Birth Rate (per 1000 population)—Using DID estimates, no association was observed between birth rates and hurricane exposure. 2003: 4.2 2004: 3.8	
Hamamatsu (2014)	Cohort January 1997–2011 Post-disaster: December 2011–June 2012	Earthquake March 11, 2011 Tohoku, Japan	Births in each prefecture	9.0 magnitude—Seismic activity intensity measured on the Japan Meteorological Agency scale as upper 5 or more in Kanto and Tohoku regions, 13 prefectures Compared to all 47 prefectures of Japan and 34 prefectures with score less than ‘upper 5’ on Japan Meteorological Agency seismic activity intensity scale	Fertility	Number of births Births in all of Japan were significantly* lower than expected for 4 of 7 post-disaster months studied (Dec 2011, Jan 2012, Apr 2012, and Jun 2012). Expected estimates were developed from a quadratic regression equation. In the disaster affected area, births were significantly lower than expected 5 out of 7 months (Dec 2011, Jan 2012, Mar–Apr 2012, Jun 2012), and in the non-disaster stricken areas, only 2 of 7 months had fewer births than expected (Apr 2012 and Jun 2012).	
Hamilton (2009)	Longitudinal administrative Pre-disaster: August 29, 2004–August 28, 2005 Post-disaster: August 29, 2005–August 28, 2006	Hurricane August 29, 2005 Gulf Coast Region, US	Births to residents of Federal Emergency Management Agency-designated disaster counties of Alabama, Louisiana, and Mississippi	91 counties with federal disaster declarations and 14 selected counties with disaster declarations within 100-mile radius of the hurricane path	Fertility	Number of births 1 year after: In 14 selected counties hardest hit 19% decline overall; 30% decrease in Louisiana, 13% decrease in Mississippi, and 6% increase in Alabama. In 91 counties studied 4% decline overall with a significant* decline in 6 counties and significant* increase in 7 counties: 12% decrease in Louisiana, 4% increase in Alabama, and 3% increase in Mississippi.	
Hapsari (2009)	Cohort, survey Before disaster and within 1 year of disaster	Earthquake May 27, 2006 Yogyakarta Province, Indonesia	Married (before disaster) women 21–49-years-old from Bantul District of Yogyakarta Province n = 450	6.2-magnitude, Richter scale—Period after earthquake considered exposed	Contraception use and access	Contraception Use—3% stopped using contraception after disaster while, 12.5% changed contraceptive method after disaster. Contraception Access—11% of pre-disaster users had difficult time accessing services after the disaster.	
Kinoshita (2016)	Mixed methods Pre-disaster: 2002–2003 Post-disaster: 2005–2006	Tsunami December 26, 2004 Aceh Province, Indonesia	Women 15-19-years-old (born 1985–1991) from Aceh Province n = 252	5 areas of province where > 10% of the population was displaced for 8 or more months after the tsunami	Fertility	Fertility Rate (per 1000 women 15–19) 2 years before: 3.5% 2 years after: 4.1%	
Kurita (2019)	Longitudinal administrative January 1, 2007–December 31, 2017	Earthquake March 11, 2011 Fukushima, Japan	Births registered in Fukushima per month divided by city population at beginning of month	All births after earthquake were considered exposed	Fertility	Birth Rate (per 100,000 population per month) Pre-disaster: 69.8 0–2 years post-disaster: 59.5 3–7 years post-disaster: 62.9 In the two years following the disaster, birth rates were significantly* lower than expected based on estimates from Poisson regression models. More than 2 years after the disaster, the birth rate returned to expected values.	
Nandi (2018)	Longitudinal administrative Pre-disaster: 1996–2000 Post-disaster: 2002–2006	Earthquake January 26, 2001 Gujarat, India	Births occurring in 1996–2000 and 2002–2006 in Gujarat, Maharashtra, Madhya Pradesh, and Rajasthan	7.7-magnitude, moment magnitude scale—Post-disaster births in Gujarat Compared to post-disaster births in Maharashtra, Madhya Pradesh, and Rajasthan	Fertility	Births—9.5% increase** in rate of childbirth among women in exposed region	
Nobles (2015)	Cohort Pre-disaster: 10 months before Post-disaster: up to 5 years after	Tsunami December 26, 2004 Aceh Province, Indonesia	Women 15–49-years-old living in Aceh Province n = 6363	Births after tsunami in 92 communities with some mortality; high (≥ 30% of residents died) or low tsunami mortality Compared to 191 communities that experienced no tsunami related mortality, in the same district as communities experiencing tsunami related mortality	Fertility	Total Fertility Rate 4 years after: Net increase of 0.7* comparing communities with some mortality to no mortality 0.5 birth per woman higher than expected in areas of high mortality	
Oyarzo (2012)	Cohort Pre-disaster: January 1–December 31, 2009 Post-disaster: March 1–December 31, 2010	Earthquake February 27, 2010 Chillan, Chile	Women delivering at Herminda Martin Clinical Hospital Pre-disaster: n = 3609 Post-disaster: n = 2553	8.8-magnitude, moment magnitude scale—All births after earthquake were considered exposed	Fertility Contraception access	Birth Rate—Compared to previous year, 9% reduction Contraceptive Access—No change	
Scapini (2021)	Longitudinal administrative 2002–2016 Pre-disaster: 2002-2009 Post-disaster: 2010–2016	Earthquake February 27, 2010 Chile	5182 registrations from 15 regions^a^	8.8-magnitude, Richter Scale 6 affected regions with modified Mercalli intensity scale level of severe or higher	Fertility	Birth Rate (per 1000 inhabitants) Pre-disaster (2004–2009): 13.85 Post-disaster (2010–2015): 12.87 Parallel trends assumption between affected and unaffected regions met. DID—Affected regions had non-significant increase in birthrate compared to unaffected regions in post-disaster period. Triple-Difference Modeling—Birth rate showed downward trend in the post-disaster period for affected and unaffected regions. Compared to the unaffected regions in the post-disaster period, the birth rate in affected regions increased* by 0.385.	
Seltzer (2017)	Longitudinal administrative 2000–2010 Pre-disaster: 2000–2004 Post-disaster: 2006–2010	Hurricane August 29, 2005 Louisiana, US	Births reported in vital statistics in New Orleans, Louisiana	Category 3 Hurricane—All births after hurricane in Orleans county and New Orleans MSA Compared to MSAs with similar population size to New Orleans and southern, costal MSAs that were not affected by hurricane	Fertility	Total Fertility Rate Asian—Constant Black—4% decrease* Hispanic—55% increase** White—5% increase* Change in TFR in post-disaster period compared to expected value based on comparable MSAs	
Tan (2009)	Cohort Pre-disaster: May 12, 2007–May 11, 2008 Post-disaster: May 12, 2008 - May 11, 2009	Earthquake May 12, 2008 Wenchaun, China	Births occurring at local hospitals in Du Jiang Yan and Peng Zhou Pre-disaster: n = 6638 Post-disaster: n = 6365	8.0-magnitude, Richter Scale—All births after earthquake were considered exposed	Fertility	Birth Rate—Constant (i.e., not a significant decrease) 4.3% decrease	
Tong (2011)	Longitudinal administrative Pre-disaster: 1994–1996 Post-disaster: 1997–2000	Flood April 1997 North Dakota, US	Births among residents giving birth in North Dakota	All births after flood were considered exposed, and six counties directly affected by flood considered most severely exposed	Fertility	Birth Rate (per 1000 population) Entire state Pre-disaster: 13.1 Post-disaster: 12.2 Most severe counties Pre-disaster: 13.9 Post-disaster: 13.0 Fertility Rate (per 1000 women 15–44) Entire state Pre-disaster: 65.3 Post-disaster: 64.0	

Constant suggests results were not statistically significant at an alpha of 0.05

*DID* difference-in-differences modeling, *GLM* generalized linear modeling, *IUD* intrauterine device, *km* kilometer, *mph* miles per hour, *MSA* metropolitan statistical area-level, *TFR* total fertility rate, *US* United States

^a^Arica and Parinacota, Tarapacá, Antofagasta, Atacama, Coquimbo, Valparaíso, Metropolitana de Santiago, Libertador General Bernardo O’Higgins, Maule, Biobío, La Araucanía, Los Ríos, Los Lagos, Aisén del General Carlos Ibáñez del Campo, and Magallanes y de la Antártica Chilena

*P < 0.05

**P < 0.01

### Fertility

Among the 18 articles describing outcomes related to fertility, five report an increase in the birth rate or fertility rate between the pre- and post-disaster study periods [3, 8, 18, 26, 27], nine reported a decrease [4, 5, 7, 9, 21–24, 28], four reported varied associations [5, 6, 19, 25], and two reported no change [20, 28]. The association varied by disaster type. Eight articles described fertility in the context of earthquakes. Most (n = 5) reported a decrease, while two described an increase, and one reported no change. In the post-disaster period, Scapini et al. [21] observed an overall decrease in birth rate compared to the pre-disaster period. However, in the post-disaster period, compared to the unaffected regions, the affected regions showed an increase in birth rate [21]. The association between fertility and hurricanes was assessed in seven articles; one reported an increase, one reported a decrease, four reported varied outcomes, and one reported no association. Both articles with an exposure of tsunami described an increase in fertility, while the article describing a flood noted a decrease. Results of the seven articles describing fertility within the United States did not show a consistent association.

### Contraception

Five studies described contraception access associated with exposure to an earthquake occurring from 2006 to 2012; three of these studies also described contraceptive use. Contraceptive access generally decreased. Bahmanjanbeh et al. [9] noted a change in annual contraception coverage from 66.9% in the year before to 64.9% in the year after the disaster. Behrman et al. [11] reported a statistically significant unmet need for contraceptives in the post-disaster period, while Djafri et al. [27] described a 20% decline in client’s self-reported perceptions of contraceptive access in the one to three months after the disaster. Hapsari et al. [12] reported 11% of pre-disaster contraceptive users had a difficult time obtaining contraceptives in the post-disaster period, while Oyarzo et al. [22] described no change in the post-disaster period. Among the three articles describing contraceptive use, two reported no change [11, 27] and one reported 3% of study participants stopped using contraception after the disaster [12].

## Discussion

In this scoping review, findings across studies varied and consistent trends in fertility following a disaster were not identified between or within disaster types. Generally, no change in contraceptive use was observed, while a general decrease in contraception access was identified. Following a disaster, infrastructure may be damaged, fuel or transportation may be unavailable, medical supplies may be depleted, and trained medical staff may be unavailable to offer provider-administered contraceptives making access to contraception difficult [10]. Results from included studies may not be comparable due to heterogeneity in study designs. This includes differences in measurement of exposure, data analysis, and study time frame relative to the disaster. Variation in results may also be attributable to differences in local, regional, and national healthcare delivery practices, and potential cultural and geographical differences in attitudes towards fertility and contraception between study settings. Future use of established reporting checklists, such as the Strengthening and Reporting of Observational Studies in Epidemiology [29] are encouraged to promote transparency in reporting and will aid in future comparisons among articles.

### Exposure measure

The measure of exposure within each disaster type was varied and future research may benefit from detailed description of how disaster exposure was measured. Disaster exposure can include the actual disaster, in addition to the threat of a disaster [30]. Additionally, consideration should be given to direct and residual disaster exposure. Therefore, multiple exposure measures can be beneficial to understanding a disaster’s impact. Exposure measures that accurately capture the populations most impacted by a disaster are needed. The misclassification of exposure measures and underreporting of disaster exposure can dampen observed associations or suggest spurious associations.

### Data analysis

Great heterogeneity of data analysis was observed among the studies included in this review. Prediction modeling may require different parameters or alternative covariates by region. While results may not be generalizable due to regional differences, the development and application of consistent data analysis methods for disaster research may improve the comparability of studies. Research describing fertility is enhanced when potential socio-demographic events and trends are accounted for, such as pre-disaster fertility decline. Disregarding the seasonality of births may mask subtle changes by month as seen in Hamamatsu et al. [24]. International evidence suggests fertility declines with an economic recession, therefore changes in the economy and migratory patterns can influence reproductive health outcomes and are important factors to consider in data analysis and interpretation [6, 24, 25]. For example, in the models developed by Evans et al. [25] standard population growth and county fixed effects were controlled for. Multiple authors used difference-in-differences models to control for county level measures and possible unmeasured ecological bias [11, 19, 21, 26]. Grabich et al. [19] compared difference-in-differences models and generalized linear models, and the resulting associations differed.

In this review, multiple studies used population data and did not have a contemporaneous non-disaster affected comparison group. Without comparing outcomes between similar exposed and unexposed populations we cannot determine if reported changes are meaningfully related to the disaster. Future research that accounts for confounders, clearly describes methodological challenges, and includes comparison groups may address these identified gaps in the literature.

### Study time frames

An appropriate post-disaster time frame is crucial for the interpretation of a study’s findings. Measuring outcomes soon after the disaster may capture immediate changes, but may not inform long-term, population level changes in fertility [25]. Oyarzo et al. [22] described birth admissions in the year prior to and 0–10 months after an earthquake. A majority of the post-disaster births were conceived prior to the disaster, therefore this short post-disaster follow-up period limits interpretation of findings for women with disaster exposure before or early in pregnancy [22]. There are analytic complexities related to disaster exposure and the timing of pregnancy (i.e., pre-pregnancy, conception, or *in utero* exposure) [20]. Therefore, disaster researchers, particularly those describing fertility, may consider multiple post-disaster assessment periods. In contrast, long-term post-disaster assessment periods may not be necessary in contraceptive use and access research. Among included articles, contraception use was determined by availability and access [12, 27]. Extending contraceptive use assessment period slightly beyond the return of contraceptive services to pre-disaster coverage may be most informative. Determining the short- and long-term changes in reproductive health following a disaster may help inform preparedness, response, and recovery interventions that better support people’s reproductive life plans.

### Overall challenges in disaster research

This scoping review included studies on natural hazard disasters worldwide to better understand the available research on the impacts to fertility and contraception. The field of disaster research is challenging due to the aforementioned heterogeneity in study design. Additionally, variations in disaster type, location, and available resources can make comparative studies difficult. The mechanisms of association between reproductive health outcomes and disasters have been difficult to determine [2, 19, 30]. Disaster literature is primarily comprised of single case studies [30]. Post-disaster research can be methodologically challenging to conduct. Studies that limit the sample to individuals in an affected geographic area may not capture outcomes among persons who are displaced due to pre-disaster evacuation or post-disaster migration [6, 11]. Data collection can be logistically difficult in a post-disaster setting and resources may be limited; delaying the timeliness of findings to inform policies and interventions. Analyses using surveillance or administrative data not originally designed for post-disaster research may be subject to unmeasured confounding and bias [19]. Articles excluded from this review for poor quality lacked clear descriptions or had poor sampling methods (Additional file 2). A convenience sample and cross-sectional study may allow for the rapid collection of data, however generalized conclusions and the direction of association become difficult to ascertain. The association between disasters and fertility is likely multifactorial, and many articles included in this review offer theoretical models to explain changes in fertility, and possibly contraception use. Examples include economic security, attachment theory, stress theory, replacement theory, and risk insurance hypothesis [3, 7, 11, 25].

Additionally, consideration may be given to the benefits and limitations of individual and aggregate level data. Individual level data may be more useful for studying behavioral changes, while aggregate data can be used to identify trends. Aggregate data are more readily available and allow for larger sample sizes but can result in exposure misclassification and suggest null results when meaningful differences are present [3, 25].

### Limitations

There are several limitations to this scoping review. Multiple studies assessed the same disaster and outcome, so study populations may have overlapped. Methods for measuring reproductive health outcomes following a disaster were not standardized. For example, across studies measuring fertility, fertility was reported as: birth rate per 1000 population, birth rate per 1000 population per month, fertility rate per 1000 women 15–44, total fertility rate, and marriage fertility rate. Few studies included unexposed comparison groups, so it is unclear if changes observed were a result of the disaster or other factors. Studies on contraception were limited by small sample sizes and post-disaster follow-up was limited to individuals using contraceptives before the disaster.

## Conclusions

This scoping review describes fertility and contraception among WRA following a disaster of natural hazards between 1989 and 2012. Among 20 articles included, variations in fertility trends and contraception use and access were observed. Based on the heterogeneity of study designs, disaster type, location, and available resources across studies the direction and magnitude of association between disasters of natural hazards and fertility remains unclear. The few studies that assessed contraception use found no change, and studies assessing contraception access generally found an overall decrease in access. This scoping review illustrates the need for more standardized research to understand the potential impacts of disasters triggered by natural hazards on fertility and contraception among WRA. Future research may benefit from clearly defined exposure measures, more robust analyses, including the exploration of factors that may influence observed associations, comparing the exposed population to a similar unexposed population, and assessing outcomes at methodical post-disaster time points.

## Supplementary information

**Additional file 1. **Data abstraction form used for citations undergoing full-text review.

**Additional file 2. **Articles meeting inclusion criteria but excluded during critical appraisal.

## Data Availability

Data sharing is not applicable to this article as no datasets were generated or analyzed during the current study.

## Abbreviations

WRA: Women of reproductive age
