# Supplementary figures and images for: Fertility and contraception among women of reproductive age following a disaster: a scoping review
Source: Reprod Health. 2022 Jun 23;19:147. doi: 10.1186/s12978-022-01436-4 (PMC9229126; doi:10.1186/s12978-022-01436-4)

**Additional file 1.** Data abstraction form used for citations undergoing full text review


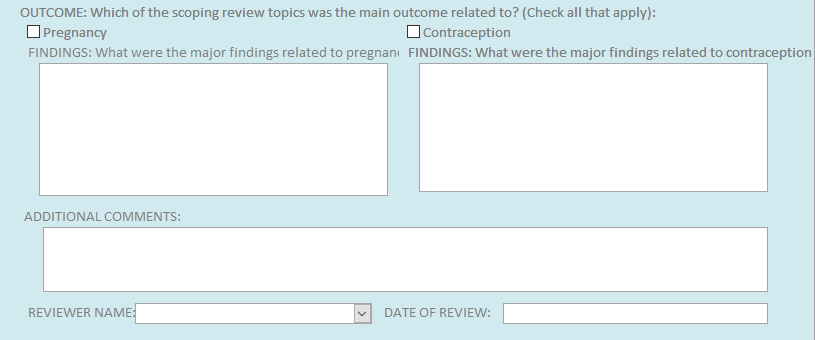

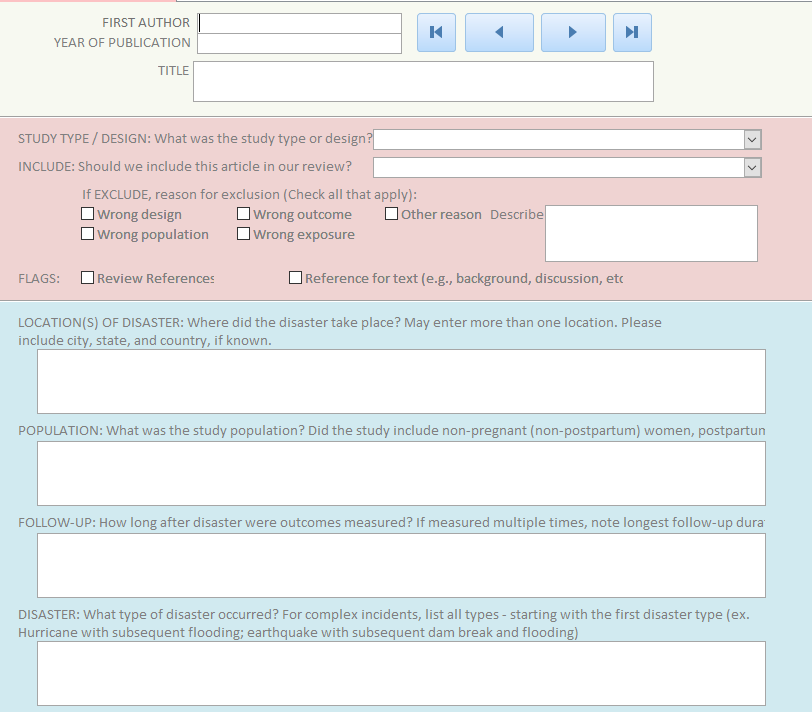

Supplement: Supplementary file 1 — Additional file 1. Data abstraction form used for citations undergoing full-text review. [file 12978_2022_1436_MOESM1_ESM.docx]
