# Supplementary material for: Fertility and contraception among women of reproductive age following a disaster: a scoping review
Source: Reprod Health. 2022 Jun 23;19:147. doi: 10.1186/s12978-022-01436-4 (PMC9229126; doi:10.1186/s12978-022-01436-4)
Supplement: Supplementary file 2 — Additional file 2. Articles meeting inclusion criteria but excluded during critical appraisal. [file 12978_2022_1436_MOESM2_ESM.docx]

**Additional file 2.** Articles meeting inclusion criteria but excluded during critical appraisal

| First author (year) | Title | Reason for poor quality assessment rating |
| --- | --- | --- |
| Bajracharya (2016) | Women of Nepal and post-earthquake humanitarian responses: an observation of three months | Unclear study design, objectives, findings, and conclusion. |
| Fukuda (1998) | Decline in sex ratio at birth after Kobe earthquake | Unclear study sampling methods and exposure measure. |
| Kissinger (2007) | The effect of the Hurricane Katrina disaster on sexual behavior and access to reproductive care for young women in New Orleans | Poor sampling methods, and unclear exposure measure. |
| Liu (2010) | A report on the reproductive health of women after the massive 2008 Wenchuan earthquake | Unclear study sampling methods and exposure measure. |
| Nasir (2018) | Prenatal exposure to shocks and early-life health: impact of terrorism and flood on birth outcomes in Pakistan | Insufficient information regarding survey response rate. |
| Westhoff (2008) | Reproductive health education and services needs of internally displaced persons and refugees following disaster | Poor sampling methods |
